# Systematic analysis of safety profile for darunavir and its boosted agents using data mining in the FDA Adverse Event Reporting System database

**DOI:** 10.1038/s41598-021-91549-w

**Published:** 2021-06-14

**Authors:** Xiaojiang Tian, Yao Yao, Guanglin He, Yuntao Jia, Kejing Wang, Lin Chen

**Affiliations:** 1 Department of Pharmacy, Chongqing Health Center for Women and Children, Chongqing, 400021 China; 2 Department of Anthropology and Ethnology, Institute of Anthropology, National Institute for Data Science in Health and Medicine, and School of Life Sciences, Xiamen University, Xiamen, 361005 China; 3 Department of Pharmacy, Children’s Hospital of Chongqing Medical University, Chongqing, 400014 China

**Keywords:** Health care, Medical research

## Abstract

This current investigation was aimed to generate signals for adverse events (AEs) of darunavir-containing agents by data mining using the US Food and Drug Administration Adverse Event Reporting System (FAERS). All AE reports for darunavir, darunavir/ritonavir, or darunavir/cobicistat between July 2006 and December 2019 were identified. The reporting Odds Ratio (ROR), proportional reporting ratio (PRR), and Bayesian confidence propagation neural network (BCPNN) were used to detect the risk signals. A suspicious signal was generated only if the results of the three algorithms were all positive. A total of 10,756 reports were identified commonly observed in hepatobiliary, endocrine, cardiovascular, musculoskeletal, gastrointestinal, metabolic, and nutrition system. 40 suspicious signals were generated, and therein 20 signals were not included in the label. Severe high signals (i.e. progressive extraocular muscle paralysis, acute pancreatitis, exfoliative dermatitis, acquired lipodystrophy and mitochondrial toxicity) were identified. In pregnant women, umbilical cord abnormality, fetal growth restriction, low birth weight, stillbirth, premature rupture of membranes, premature birth and spontaneous abortion showed positive signals. Darunavir and its boosted agents induced AEs in various organs/tissues, and were shown to be possibly associated with multiple adverse pregnant conditions. This study highlighted some novel and severe AEs of darunavir which need to be monitored prospectively.

## Introduction

The burden of morbidity and mortality associated with human immunodeficiency virus (HIV) infection has become a serious public health problem globally^1^. WHO and most national guidelines recommended all people living with HIV to start antiretroviral therapy (ART) irrespective of clinical or immune status^1,2^. Earlier initiation of ART has led to an overall improvement in disease control, and the annual number of people dying from HIV-related causes has declined by 60% since the peak in 2004^3^. However, the increasing use of antiretroviral agents has raised potential safety concerns of these drugs which need to be systemically analyzed.

Darunavir, a nonpeptidic inhibitor of the HIV-1 protease with potent activity against resistant virus, was initially approved by the Food and Drug Administration (FDA) in 2006 for the treatment of antiretroviral-experienced adults, and later for naive adults. It must be co-administered with a boosting agent, either ritonavir or cobicistat. In 2008, FDA required labeling change of darunavir, warning the safety issues. In recent years, multiple studies reported the adverse events (AEs) of darunavir-containing agents related to hepatic^4^ and skin system^5^. In addition, darunavir was considered a preferred protease inhibitor (PI) for pregnant females living with HIV by the Health and Human Service (HHS) panel, its safety information during pregnancy was still under ongoing monitoring^6^. In 2015, the antiretroviral pregnancy registry steering committee suggested that prenatal exposure to PIs can lead to increased risk of miscarriage and low birth weight^7^. Nevertheless, clinical data on pregnancy outcomes and fetal safety after darunavir exposure during pregnancy are limited.

The primary aim of this pharmacovigilance study was to characterize the safety profile of darunavir-containing agents relating to various organ systems, moreover, evaluate the perinatal outcomes in HIV mothers exposed to darunavir during pregnancy using data-mining of FDA Adverse Event Reporting System (FAERS).

## Results

### Descriptive analysis

During the study period, a total of 11,170,959 reports were submitted to FAERS, of which 10,756 reports and 27,234 AEs for darunavir and its boosted agents, making each report contributing 2.53 AEs in average. Table 1 described the characteristics of AE reports submitted for darunavir. Higher rate of male patients (n = 5898, 54.47%) was reported than female patients (n = 3111, 28.92%); 49.31% of the AEs occurred in people aged 18–60 years; serious adverse events (SAEs) accounted for a relatively high proportion (41.47%), with hospitalization and prolonged hospitalization being the most reported outcome (32.57%).

**Table 1:** Characteristics of adverse event reports submitted for darunavir and its boosted agents.

	N. of reported AEs	Ratio (%)
**Gender**		
Male	5859	54.47
Female	3111	28.92
Unknown	1786	16.61
**Age group, (y)**		
< 18	477	4.43
18–44	2490	23.15
45–64	2814	26.16
65–74	342	3.18
≥ 75	101	0.94
Unknown	4532	42.13
**Reporters**		
Doctors	3950	36.72
Pharmacists	1022	9.50
Other medical staff	3628	33.73
Lawyers	18	0.17
Consumers	1836	17.07
Unkown	302	2.81
**SAEs**^**a**^		
Death	102	0.95
Life-threatening	526	4.89
Hospitalization (initial or prolonged)	3503	32.57
Disability	329	3.06

^a^Serious adverse events.

### Signals of SDR and BCPNN

When AEs were classified with System Organ Class (SOC) of Medical Dictionary of Regulatory Activities (MedDRA), the positive signals detected by the three algorithms were consistent, involving 11 organ systems: liver, kidney, metabolic and nutritional system, endocrine system, eye, cardiac system, musculoskeletal system, nervous system, skin, gastrointestinal tract, and perinatal periods (Table 2). Disproportionate reporting (SDR) and Bayesian confidence propagation neural network (BCPNN) of the standardized MedDRA queries (SMQs) analysis were summarized in Table 3. 13 SMQs emerged with statistical significancy.

**Table 2:** Involved systems and signal strength for darunavir and its boosted agents based on system organ class.

SOC^a^	ROR^b^ (95%CI)	PRR^c^ (*χ*^2^)	IC^d^ (IC-2SD)
Hepatobiliary disorders	3.03 (2.88–3.20)	2.72 (1882.57)	1.44 (1.41)
Renal and urinary disorders	2.45 (2.33–2.57)	2.19 (1341.86)	1.13 (1.10)
Metabolism and nutrition disorders	2.44 (1.35–1.56)	2.41 (103.75)	0.50 (0.47)
Endocrine disorders	13.92 (12.43–15.59)	13.55 (3585.85)	3.69 (3.65)
Eye disorders	21.44 (18.63–24.66)	21.05 (3783.56)	4.24 (4.20)
Cardiac disorders	2.03 (1.01–2.24)	2.04 (4.05)	0.04 (0.01)
Musculoskeletal and connective tissue disorders	7.96 (7.33–8.65)	7.58 (3374.00)	2.90 (2.87)
Nervous system disorders	7.16 (6.48–7.90)	6.93 (2061.09)	2.76 (2.73)
Skin and subcutaneous tissue disorders	10.90 (10.28–11.57)	9.75 (9877.79)	3.26 (3.23)
Gastrointestinal disorders	10.10 (9.58–10.65)	8.71 (11,314.98)	3.11 (3.08)
Pregnancy, puerperium and perinatal conditions	12.63 (12.05–13.24)	10.26 (18,528.56)	3.34 (3.31)

^a^SOC System organ class.

^b^ROR reporting odds ratio. The lower limits of the 95% CI of the ROR greater than 1 indicated statistically significant RORs.

^c^PRR proportional reporting ratio. PRR and *χ*^2^ greater than 2 and 4 respectively indicated statistically significant PRRs.

^d^Information component. The signal was statistically significant when IC-2SD > 0.

**Table 3:** Signal strength for darunavir and its boosted agents at the SMQ level in FAERS.

SMQs^a^	ROR^b^ (95%CI)	PRR^c^ (*χ*^2^)	IC^d^ (IC-2SD)
Cholestasis and jaundice of hepatic origin	4.73 (4.25–6.23)	4.02 (4524.3)	1.65 (1.60)
Proteinuria	2.16 (1.56–4.01)	2.54 (891.2)	1.19 (1.12)
Lipodystrophy	2.78 (2.50–5.94)	2.14 (1562.4)	1.02 (1.01)
Hyperglycaemia/new onset diabetes mellitus	1.41 (1.02–2.31)	1.36 (2301.5)	0.92 (0.89)
Severe cutaneous adverse reactions	12.45 (10.87–13.23)	10.98 (12,541.2)	3.72 (3.54)
Central nervous system vascular disorders	1.56 (1.11–1.97)	1.25 (569.7)	0.51 (0.49)
Noninfectious diarrhoea	6.84 (6.12–7.45)	6.08 (5423.1)	2.71 (2.66)
Dyslipidaemia	2.39 (1.52–3.41)	2.22 (532.3)	1.24 (1.01)
Acute pancreatitis	4.12 (3.97–4.85)	4.05 (5213.4)	2.87 (2.69)
Angioedema	1.29 (1.17–1.56)	1.21 (2125.6)	0.56 (0.52)
Gastrointestinal nonspecific inflammation and dysfunctional conditions	3.29 (3.07–4.85)	3.14 (4524.3)	1.82 (1.79)
Hypersensitivity	8.56 (7.41–9.52)	8.41 (9541.2)	3.02 (2.98)
Pregnancy, labour and delivery complications and risk factors	13.25 (12.89–15.68)	12.98 (12,679.8)	3.18 (3.09)

^a^SMQs standardized MedDRA queries.

^b^ROR reporting odds ratio. The lower limits of the 95% CI of the ROR greater than 1 indicated statistically significant RORs.

^c^PRR proportional reporting ratio. PRR and *χ*^2^ greater than 2 and 4 respectively indicated statistically significant PRRs.

^d^Information component. The signal was statistically significant when IC-2SD > 0.

Further analyses conducted at the PT level revealed 40 suspicious signals, 20 of which were not included in the label. Among them, 6 suspicious signals were generated in hepatobiliary system, including hepatocyte injury, hyperbilirubinemia, cholestasis, etc.; 6 signals in kidney and urinary system, including renal tubular necrosis, decreased glomerular filtration rate, and proteinuria, ect; 3 in metabolism and nutrition system: hypertriglyceridaemia, hypercholesterolaemia and hypokalaemia; 1 in cardiovascular system: blood creatine phosphokinase increased; 1 in musculoskeletal system: rhabdomyolysis; 4 in skin and subcutaneous tissue: rash generalized, pruritus, dermatitis exfoliative, and Stevens-Johnson Syndrome; 4 in gastrointestinal system: diarrhea, gastrointestinal disorder, oesophageal candidiasis, and acute pancreatitis. It was worth noting that darunavir-containing agents can induce progressive ophthalmoplegia, lipodystrophy acquired, mitochondrial toxicity, adrenal suppression and other severe high strength signals (Table 4).

**Table 4:** Signal strength for darunavir and its boosted agents based on PT level in FAERS.

PTs^a^	N^b^	ROR^c^ (95%CI)	PRR^d^ (*χ*^2^)	IC^e^ (IC-2SD)	Label^f^
**Hepatobiliary disorders**					
Hepatocellular injury	105	12.00 (9.89–14.56)	11.89 (1025.76)	3.42 (3.38)	Yes
Liver function test abnormal	88	3.93 (3.18–4.85)	3.90 (186.81)	1.91 (1.88)	Yes
Hepatic enzyme increased	147	3.80 (2.85–5.05)	3.78 (90.29)	1.83 (1.80)	Yes
Jaundice	72	3.28 (2.60–4.13)	3.26 (110.46)	1.66 (1.62)	No
Cholestasis	78	7.61 (6.09–9.52)	7.57 (435.14)	2.80 (2.76)	No
Hyperbilirubinaemia	46	7.62 (5.70–10.19)	7.60 (255.16)	2.73 (2.68)	No
**Renal and urinary disorders**					
Acute kidney injury	164	2.53 (2.17–2.95)	2.51 (148.32)	1.32 (1.28)	Yes
Renal impairment	150	3.49 (2.97–4.10)	3.46 (259.46)	1.32 (1.29)	Yes
Blood creatinine increased	116	2.84 (2.36–3.41)	2.82 (134.12)	1.49 (1.44)	Yes
Glomerular filtration rate decreased	35	6.86 (4.92–9.57)	6.84 (167.83)	2.55 (2.50)	Yes
Proteinuria	53	5.44 (4.40–4.57)	5.75 (202.23)	2.40 (2.35)	No
Renal tubular necrosis	37	6.93 (5.01–9.58)	6.91 (180.08)	2.57 (2.53)	No
**Metabolism and nutrition disorders**					
Hypertriglyceridaemia	49	15.81 (11.92–20.98)	15.75 (662.25)	3.59 (3.53)	Yes
Hypercholesterolaemia	36	7.31 (5.26–10.15)	7.29 (187.79)	2.63 (2.58)	Yes
Hypokalaemia	40	1.57 (1.15–2.15)	2.57 (7.78)	0.63 (0.60)	No
**Endocrine disorders**					
Hyperglycaemia	60	2.78 (3.16–3.59)	2.77 (66.29)	1.43 (1.39)	Yes
Adrenal insufficiency	54	11.89 (9.08–15.56)	11.83 (519.06)	3.29 (3.24)	No
Adrenal suppression	28	44.60 (30.54–65.14)	44.49 (1100.12)	4.13 (4.02)	No
**Eye disorders**					
Diplopia	63	4.24 (3.31–5.43)	4.22 (151.11)	2.00 (1.97)	No
Eyelid ptosis	59	10.43 (8.06–13.49)	10.38 (486.02)	3.15 (3.11)	No
Progressive external ophthalmoplegia	49	1761.17 (1112.25–2788.69)	1753.15 (31,253.87)	3.54 (3.21)	No
**Cardiac and vascular disorders**					
Blood creatine phosphokinase increased	73	3.47 (2.76–4.37)	3.46 (124.84)	1.74 (1.70)	Yes
**Musculoskeletal and connective tissue disorders**					
Rhabdomyolysis	76	3.00 (2.39–3.76)	2.99 (98.36)	1.54 (1.51)	YeS
**Nervous system disorders**					
Nervous system disorder	45	1.76 (1.31–2.36)	3.68 (235.32)	0.79 (0.76)	No
Neuropathy peripheral	119	2.50 (2.08–3.00)	2.48 (103.91)	1.29 (1.26)	No
**Skin and subcutaneous tissue disorders**					
Rash generalised	74	2.34 (1.87–2.95)	2.34 (55.32)	1.20 (1.17)	Yes
Pruritus	44	2.55 (1.15–2.09)	2.32 (54.21)	0.61 (0.58)	Yes
Dermatitis exfoliative	53	10.26 (7.83–13.46)	10.22 (46.74)	3.11 (3.07)	Yes
Stevens-Johnson Syndrome	47	3.11 (2.33–4.14)	3.10 (64.68)	1.57 (1.53)	Yes
**Gastrointestinal disorders**					
Diarrhoea	405	1.21 (1.12–1.37)	2.23 (18.11)	0.30 (0.27)	Yes
Gastrointestinal disorder	84	1.71 (1.38–2.12)	2.71 (24.03)	0.76 (0.73)	Yes
Oesophageal candidiasis	37	16.02 (11.57–22.18)	15.97 (496.77)	3.50 (3.44)	No
Pancreatitis acute	59	4.21 (3.26–8.44)	4.19 (139.96)	1.26 (1.23)	Yes
**Others**					
Virologic failure	254	117.48 (103.06–133.93)	114.73 (256.88)	6.02 (6.14)	No
Drug resistance	224	17.31 (15.15–19.78)	16.97 (3301.72)	3.97 (3.93)	No
Lipodystrophy acquired	153	137.98 (116.46–163.48)	136.03 (18,013.82)	6.08 (6.00)	No
Viral mutation identified	75	52.98 (41.98–66.86)	52.62 (3565.80)	4.93 (4.85)	No
Mitochondrial toxicity	68	171.92 (132.93–222.34)	107.84 (9713.13)	5.56 (5.42)	No
Angioedema	42	1.47 (1.09–1.99)	2.47 (15.84)	0.54 (0.51)	Yes
Erectile dysfunction	29	2.03 (1.41–2.93)	2.03 (14.19)	0.97 (0.94)	No

^a^PT: preferred terms.

^b^Number of patients with adverse events.

^c^ROR reporting odds ratio. The lower limits of the 95% CI of the ROR greater than 1 indicated statistically significant RORs.

^d^PRR proportional reporting ratio. PRR and *χ*^2^ greater than 2 and 4 respectively indicated statistically significant PRRs.

^e^Information component. The signal was statistically significant when IC-2SD > 0.

^f^Whether adverse events are mentioned in the drug label or not.

Among pregnant women, umbilical cord abnormality, foetal growth restriction, low birth weight baby, stillbirth, premature rupture of membrane, premature baby and abortion spontaneous showed positive signals (Table 5). When detected separately, signals of abortion spontaneous and foetal growth restriction for darunavir/cobicistat were positive, and premature baby for darunavir/ritonavir positive (Table 6).

**Table 5:** Signal strength of pregnancy, puerperium and perinatal conditions for darunavir and its boosted agents.

PT^a^	N^b^	ROR^c^ (95%CI)	PRR^d^ (*χ*^2^)	IC^e^ (IC-2SD)
Foetal exposure during pregnancy	813	20.73 (19.28–22.28)	19.24 (13,836.29)	4.21 (4.18)
Premature baby	272	16.50 (14.62–18.63)	16.11 (3789.36)	3.91 (3.88)
Abortion spontaneous	269	10.87 (9.63–12.28)	10.62 (2317.55)	3.35 (3.31)
Foetal growth restriction	105	37.37 (30.73–45.45)	37.02 (3519.26)	4.75 (4.69)
Low birth weight baby	95	26.54 (21.13–32.56)	26.31 (2232.29)	4.35 (4.30)
Stillbirth	70	22.59 (17.81–28.65)	22.45 (1383.80)	4.09 (4.03)
Premature rupture of membranes	38	17.20 (12.48–23.71)	17..14 (552.58)	3.58 (3.52)
Umbilical cord abnormality	29	71.32 (48.94–103.95)	71.13 (1811.59)	4.38 (4.25)

^a^PT: Preferred terms.

^b^Number of patients with adverse events.

^c^ROR reporting odds ratio. The lower limits of the 95% CI of the ROR greater than 1 indicated statistically significant RORs.

^d^PRR proportional reporting ratio. PRR and *χ*^2^ greater than 2 and 4 respectively indicated statistically significant PRRs.

^e^Information component. The signal was statistically significant when IC-2SD > 0.

**Table 6:** Signal strengthof pregnancy, puerperium and perinatal conditions for darunavir/cobicistat and darunavir/ritonavir.

PT^a^	Darunavir/cobicistat				Darunavir/ritonavir			
	N^b^	ROR^c^ (95% CI)	PRR^d^ (*χ*^2^)	IC^e^ (IC-2SD)	N	ROR (95% CI)	PRR (X2)	IC (IC-2SD)
Foetal exposure during pregnancy	54	18.3 (13.88–24.12)*	17.11 (805.55)*	3.72 (2.30)*	204	1.30 (1.14–1.50)*	1.30 (14.12)*	2.33 (2.30)*
Premature baby	1	0.79 (0.11–5.65)	0.79 (0.05)	0.18 (0.28)	123	1.98 (1.66–2.37)*	1.98 (58.22)*	0.38 (0.36)*
Abortion spontaneous	10	5.39 (2.85–4.09)*	5.33 (31.04)*	1.93 (1.83)*	80	0.86 (0.69–1.07)	0.86 (1.71)	0.97 (0.94)
Foetal growth restriction	25	120.7 (80.93–180.08)*	116.93 (2736.87)*	4.42 (4.30)*	16	1.50 (0.92–2.45)	1.50 (2.17)	0.22 (0.19)
Low birth weight baby	1	3.69 (0.52–26.20)	3.68 (0.19)	0.65 (0.54)	6	0.44 (0.20–1.00)	0.44 (3.62)	1.05 (1.00)
Stillbirth	1	4.28 (0.60–30.46)	4.28 (0.30)	0.70 (0.58)	9	0.78 (0.40–1.49)	0.78 (0.38)	0.33 (0.28)
Premature rupture of membranes	0	–	–	–	9	1.10 (0.57–2.11)	1.10 (0.01)	0.16 (0.06)
Umbilical cord abnormality	0	–	–	–	1	0.63 (0.09–4.48)	0.63 (0.00)	0.37 (0.24)

^a^PT: preferred terms.

^b^Number of patients with adverse events.

^c^ROR reporting odds ratio. The lower limits of the 95% CI of the ROR greater than 1 indicated statistically significant RORs.

^d^PRR proportional reporting ratio. PRR and *χ*^2^ greater than 2 and 4 respectively indicated statistically significant PRRs.

^e^Information component. The signal was statistically significant when IC-2SD > 0.

*Statistically significant.

## Discussion

As far as we know, this is the first comparative safety study on FAERS that aimed to assess the reported AEs of darunavir and its boosted agents. Overall, three main findings emerged: (1) AEs related to darunavir exposure involve various organs or tissues. We found statistically significant signals in the liver, kidney, metabolic and nutritional system, endocrine system, eye, cardiac system, musculoskeletal system, nervous system, skin, and gastrointestinal tract when classified with SOC, and 13 SMQs involving various systems emerged. (2) Strongly positive signals related to mitochondrial toxicity (ROR = 171.92, PRR = 136.03, χ^2^ = 9713.13, IC-2SD = 5.42) and eye disorders (included diplopia, eyelid ptosis, and progressive external ophthalmoplegia) were revealed for the very first time. (3) Signals for adverse pregnancy outcomes were detected in our study, which highlights its safety concern during pregnancy.

Studies indicated that some degree of serum aminotransferase elevations occurred in a high proportion of patients with darunavir^4,8^ Our study uncovered positive signals for hepatocellular injury and elevation in serum hepatic enzymes which were consistent with the previous findings. Apart from hepatocellular injury, we also found darunavir can induce increased bilirubin, cholestasis, and jaundice which were not observed in clinical studies. Yancheva^9^ reported a case of darunavir-related cholestatic hepatitis in an HIV patient in the third year of his antiretroviral therapy. The toxic intermediates may be the cause of some liver injury. It is worth noting that, except for hepatocellular injury, cholestasis should also be monitored when darunavir is prescribed.

In our study, 6 positive signals of the renal and urinary system were detected (AKI, renal impairment, blood creatinine increased, glomerular filtration rate decreased, proteinuria, and renal tubular necrosis). It was showed that cobicistat inhibits tubular secretion of creatinine without affecting actual glomerular function^10^. This should be considered when interpreting changes in creatinine. Besides, our study uncovered an association of darunavir with rhabdomyolysis, which might be one of the causes of kidney injury. On the other hand, we should take caution explaining the significant signal of darunavir in renal injury, since HIV-associated nephropathy is one of the complications in advanced HIV disease, the main manifestations of which were heavy proteinuria and a decline in kidney function^11^. In accord with this assumption, drug resistance and treatment failure are significantly noted in the analysis, which implicated the occurrence of advanced HIV disease.

Our findings showed a disproportionate association with hypertriglyceridemia, hypercholesteremia, and hypokalemia. It was consistent with the previous findings that exposure to certain PIs can cause an adverse change in the lipid profile^12^. In a previous study, 15% of patients with boosted darunavir developed elevated triglyceride levels compared with 7% percent in the comparator PI arms^13^. The signal of lipodystrophy, which has been associated with abnormalities in glucose and lipid metabolism, was extremely strong in our study. Lipodystrophy can be manifested as lipoatrophy or fat accumulation, and 10–80% of HIV patients developed these changes^14,15^. Data suggested that exposure to certain nucleoside reverse transcriptase inhibitors (NRTIs) is the major factor of lipoatrophy^15^. Some studies showed that PIs may act synergistically with NRTIs^16^, and therapy with PIs alone does not appear to lead to lipoatrophy^17^. Fat accumulation was initially thought to be lead by the use of PIs^18^. A study showed that body fat tissue increased in patients on darunavir/ritonavir monotherapy and darunavir/ritonavir plus NRTIs, with no difference between the arms^19^.

Hyperglycemia is another positive signal identified in our study. Animal experiments and clinical trials of PIs have demonstrated insulin resistance with these agents^20^. One possible explanation is that PIs can direct down-regulation of the glucose transporter-4, the major transporter of glucose into fat cells, and cardiac and skeletal muscle^21^. Since HIV-positive persons are at increased risk for premature cardiovascular disease (CVD)^22^, dyslipidemia and hyperglycemia caused by darunavir can adversely affect the risk factors for CVD. However, the association between darunavir or atazanavir and increased risk of myocardial infarction or stroke has not been established which was seen with other PIs^23^.

Hypokalemia is common in AIDS inpatients, usually due to AIDS-related gastrointestinal complications^24^. Hypokalemia directly caused by darunavir has not been reported, but it was suggested that diarrhea and vomiting were the most common adverse reactions of darunavir^25^, which we presumed might to be the cause of hypokalemia.

Adrenal suppression and dysfunction were found related to the use of darunavir-containing agents. The possible explanation may be due to drug-drug interaction of pharmacokinetic boosters with exogenous glucocorticoids^26^. Glucocorticoids, including non-systemic preparations, were widely used in HIV patients for non-AIDS-related conditions^27^. Iatrogenic Cushing's syndrome can result from the co-administration of ritonavir or cobicistat and synthetic glucocorticoids given by any route^28,29^. The effects of these boosters on cytochrome P450 lead to prolongation of the half-life of the latter. The resultant high plasma levels of glucocorticoid cause Cushing's syndrome and secondary adrenal insufficiency.

Our study revealed an association of mitochondrial toxicity and darunavir that has not been reported previously. Mitochondrial toxicity has been recognized as a major adverse effect with NRTIs^30^, but not darunavir or other PIs. The clinical expression of mitochondrial disorders is extremely variable, and organs and tissues highly related to oxidative phosphorylate (muscles and neuro for instance) are mostly easily involved. Muscle symptoms included exercise intolerance, fatigue, muscle weakness, elevated serum creatine kinase, myalgia, or less often, rhabdomyolysis^31^. It is not surprising to find that, increased creatine phosphokinase and rhabdomyolysis are both positive signals. Mitochondrial toxicity might be a possible explanation for the cause of these AEs.

Other novel AEs inferred to be associated with mitochondrial toxicity were eye disorders^32^, which included diplopia, eyelid ptosis, and progressive external ophthalmoplegia (PEO). Among them, the signal of PEO showed a significantly high strength (ROR = 1761.17, PRR = 1753.15, IC = 3.54). PEO is a myopathic alteration of slow progression which affects extrinsic ocular muscles; ptosis of the eyelid being the most characteristic sign. Some cases progress to eye immobilization. PEO is one of the clinical phenotypes of mitochondrial myopathies^33^. We speculated that darunavir induced eye disorders through mitochondrial toxicity, although the relationship has to be confirmed with rigorous studies.

There is little doubt that mitochondrial toxicity is the major cause of NRTIs-induced myopathy and neuropathy^34^, and we can't help but speculate the newly found AEs with nervous system disorders (neuropathy and peripheral neuropathy) of darunavir in our study might also be related to mitochondrial toxicity. However, this speculation needs to be further investigated.

We found generalized rash, pruritus, exfoliative dermatitis and Stevens-Johnson Syndrome (SJS) were positive signals in the skin and subcutaneous tissue. In clinical trials, rash occurred in 16% of subjects, which were generally mild-to-moderate^35^. Severe skin rash, including erythema multiforme and SJS has also been reported^36^. The discontinuation rate due to rash was 0.3%^25^. The incidence of SJS is 100-fold higher among HIV individuals^37^. The reasons for the susceptibility are not fully understood, although exposure to multiple medications may contribute^38^. Our study brought to the forefront again the risk of severe adverse skin reactions caused by darunavir.

Our study identified diarrhea, gastrointestinal disorder, esophageal candidiasis, and acute pancreatitis as positive signals in the gastrointestinal system. Diarrhea is one of the most commonly reported adverse reactions for darunavir (10%)^39^. Esophageal candidiasis, which is typically seen in patients with HIV who have advanced immunosuppression, may not be directly related to the administration of darunavir, but rather to the failure of antiviral therapy^40^. Acute pancreatitis induced by darunavir-based ARTs has been reported previously^41^. Hypertriglyceridemia and hypercholesteremia related to darunavir may play a role. Besides, it was suggested that mitochondrial toxicity may be the cause of NRTI-induced pancreatitis^42^, the possibility cannot be ruled out that acute pancreatitis is one of the manifestations of darunavir-induced mitochondrial toxicity.

PIs have been a key component of HIV therapy in pregnant women. In 2016, darunavir/ritonavir replaced lopinavir/ritonavir as a recommended agent due to its potent antiretroviral activity and a lower rate of causing lipid abnormality^43^. The fetal transfer rate of darunavir was 12–16%, and a mean concentration of 132 ± 32 ng/mL was identified in the fetal compartment^44^. Such exposure may provide the benefit of pre-exposure prophylaxis, but it could also lead to toxicity. Although teratogenicity has not been identified in animal studies^25^, no well-designed controlled trials have been performed in humans. The antiviral pregnancy registry reported that the risk of birth defects did not increase following darunavir exposure^45^, and darunavir could even play a protective role in the development of microcephaly^46^. Our study showed positive signals for darunavir in terms of premature baby, spontaneous abortion, foetal growth restriction, low birth weight baby, stillbirth, premature rupture of membranes, and umbilical cord abnormality. In the previous studies, preterm birth and low birth weight were the most commonly reported adverse events after pregnancy exposure to PIs^47,48^. One study suggested that prematurity was independently associated with ritonavir-boosted PI therapy during pregnancy^49^. We further detected signals for darunavir/ritonavir and darunavir/cobicistat respectively, identifying positive signal for darunavir/ritonavir only in prematurity, and darunavir/cobicistat in abortion spontaneous and feotal growth restriction. The result further verified that preterm birth may be more associated with ritonavir. Since the combination of darunavir/cobicistat is not currently recommended during pregnancy due to a lack of safety data for cobicistat^43^, the difference of these two combinations for the offspring need to be further studied.

Despite some steps were taken to make the results more reliable, the following limitations of our study need to be noticed: (1) we derived ROR, PRR, and IC values based on the reported frequency of drug-event combinations, and were adjusted based on the rates reported by other drugs and the rates of all other AEs reported for the studied drug. The value indicated an increased risk of AE reporting and not a risk of AE occurrence. (2) The FAERS database is subject to various biases such as under-reporting, over-reporting, duplicates, unverified source of submitted data, missing information, misspelling, etc. (3) Certainty that the drug is in fact responsible for the reported event is absent. This is particularly true for antiretroviral agents since HIV infection per se can induce a higher risk of multi-system complications and are generally treated with a combination of antiviral drugs. The absence of previous exposure to other HIV treatments as well as the stage of disease progression makes it difficult to evaluate the influence of other antiviral drugs and the disease. (4) Except for pregnancy and perinatal conditions, the signal mining was not carried out separately for darunavir, darunavir/ritonavir, and darunavir/cobicistat, making it impossible to distinguish AEs resulted from darunavir and boosters.

## Conclusions

The safety profile of darunavir containing agents was reviewed using the AEs submitted to the FAERS. Base on the 10,756 reports, AEs with darunavir and its boosted agents took place in many organs/tissues. An association related to mitochondrial toxicity was identified and was presumed to be associated with the occurrence of AEs in multiple systems (eye, muscle, nerve, etc.). Darunavir was shown to be possibly associated with multiple adverse pregnancy conditions. The usefulness of pharmacovigilance research should be corroborated with the real-world FAERS data; however, further clinical trials and real-world study are required to confirm our findings.

## Methods

### Data sources

The data for this study were retrieved from the public release of the FAERS database, which adheres to the international safety reporting guidance issued by the International Conference on Harmonisation (ICH E2B). AEs are coded using preferred terms (PTs) in the Medical Dictionary for Regulatory Activities (MedDRA) terminology. Currently, FAERS comprises more than 12 million reports gathered worldwide. These post-marketing reports contain relevant anonymised information relating to the AEs, include: (1) Demographic and administrative information and the initial report image ID number; (2) Drug information from the case reports; (3) Reaction information from the reports; (4) Patient outcome information from the reports, etc. We conducted a retrospective pharmacovigilance study using data from the FAERS database covering the period from July 2006 to December 2019 through the OpenVigil FDA platform. To ensure data integrity, AE reports for “darunavir”, “darunavir/ritonavir” or “darunavir/cobicistat” were analyzed. The reports were included only if the drug was primary and secondary suspected. We removed duplicated records according to the FDA’s recommendations by selecting the latest FDA_DT when the CASE_ID and FDA_DT were the same, and excluded reports with more than three differences. We also excluded reports with more than 3 items of missing information.

### Definition of AEs

SDR and BCPNN were performed using all existent narrow SMQs and SOCs. Further analysis on PT levels was conducted using the same method. Two researchers classified the AEs reports in terms of SMQs, SOCs and PTs, and collected clinical characteristics of the patient, including gender, age, AE outcome, and type of reporter, respectively. Death, life-threatening adverse drug experience, inpatient/prolonged hospitalization, and significant disability/incapacity were defined as SAEs.

### Data mining algorithm and statistical analysis

Descriptive analyses were conducted to summarize the clinical characteristics of the patients with darunavir-associated AEs collected from the FAERS database. In this study, the signals of SDR and BCPNN were generated by calculating the reporting odds ratio (ROR), proportional reporting ratio (PRR), information component(IC). These methods were based on two-by-two contingency (Table 7). An association between drug and an AE was identified when all the three algorithms were positive. The equations and criteria for the algorithms are shown in Table 8^50–52^. The analyses were conducted using the Microsoft EXCEL 2010 and SPSS 13.0 statistical software.

**Table 7:** Two-by-two contingency table for disproportionality analyses.

	Reports with the target AEs	All other AEs	Total
Reports with the target drug	a	b	a + b
All other drugs	c	d	c + d
Total	a + c	b + d	a + b + c + d

**Table 8:** Summary of major algorithms used for signal detection.

Algorithms	Equation	Criteria
ROR	ROR = (a/b)/(c/d)	95% CI > 1, N ≥ 3
PRR	PRR = (a/(a + c))/(b/(b + d))	PRR ≥ 2, χ^2^ ≥ 4, N ≥ 3
BCPNN	IC = log^2^a (a + b + c + d)/((a + c) (a + b))	IC-2SD > 0
